# ENT2 facilitates brain endothelial cell penetration and blood-brain barrier transport by a tumor-targeting anti-DNA autoantibody

**DOI:** 10.1172/jci.insight.145875

**Published:** 2021-07-22

**Authors:** Zahra Rattray, Gang Deng, Shenqi Zhang, Anupama Shirali, Christopher K. May, Xiaoyong Chen, Benedette J. Cuffari, Jun Liu, Pan Zou, Nicholas J.W. Rattray, Caroline H. Johnson, Valentina Dubljevic, James A. Campbell, Anita Huttner, Joachim M. Baehring, Jiangbing Zhou, James E. Hansen

**Affiliations:** 1 Department of Therapeutic Radiology and; 2 Department of Neurosurgery, Yale School of Medicine, New Haven, Connecticut, USA.; 3 Yale School of Public Health, New Haven, Connecticut, USA.; 4 Yale Cancer Center, New Haven, Connecticut, USA.; 5 Patrys Ltd., Melbourne, Australia.; 6 Department of Pathology and; 7 Department of Neurology, Yale School of Medicine, New Haven, Connecticut, USA.

**Keywords:** Autoimmunity, Oncology, Brain cancer, Breast cancer, Immunoglobulins

## Abstract

The blood-brain barrier (BBB) prevents antibodies from penetrating the CNS and limits conventional antibody-based approaches to brain tumors. We now show that ENT2, a transporter that regulates nucleoside flux at the BBB, may offer an unexpected path to circumventing this barrier to allow targeting of brain tumors with an anti-DNA autoantibody. Deoxymab-1 (DX1) is a DNA-damaging autoantibody that localizes to tumors and is synthetically lethal to cancer cells with defects in the DNA damage response. We found that DX1 penetrated brain endothelial cells and crossed the BBB, and mechanistic studies identify ENT2 as the key transporter. In efficacy studies, DX1 crosses the BBB to suppress orthotopic glioblastoma and breast cancer brain metastases. ENT2-linked transport of autoantibodies across the BBB has potential to be exploited in brain tumor immunotherapy, and its discovery raises hypotheses on actionable mechanisms of CNS penetration by neurotoxic autoantibodies in CNS lupus.

## Graphical abstract

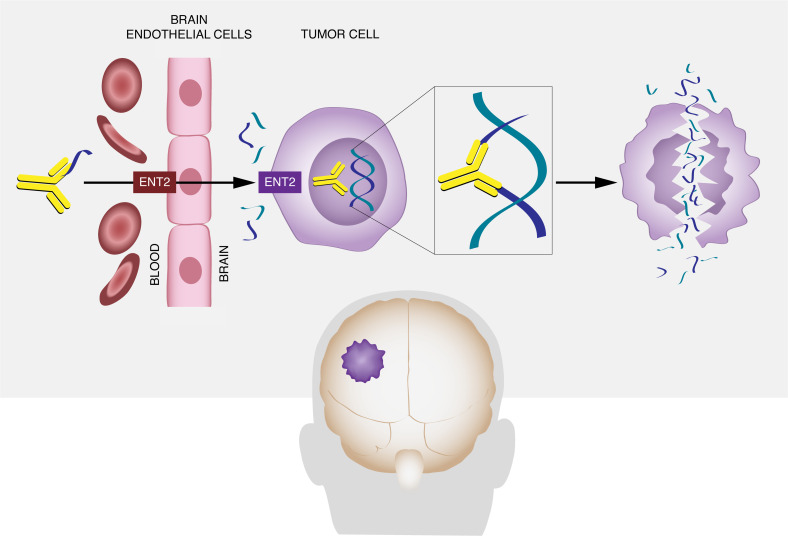

## Introduction

Antibodies have revolutionized cancer therapy, but the blood-brain barrier (BBB) limits their use against brain tumors (1, 2). Local BBB perturbations at tumors are generally insufficient to allow nonspecific uptake of macromolecules into the brain (3), and delivery of antibodies to the CNS remains challenging despite advances in methods of convection-enhanced delivery and BBB disruption (4). Techniques to carry linked antibodies across the BBB by receptor-mediated transcytosis are under development (5, 6), but widespread distribution of the relevant receptors may lead to off-target effects. Alternative approaches to transporting antibodies across the BBB are needed.

Some autoantibodies reactive against host DNA penetrate live cells, and modified cell-penetrating autoantibodies are in development for use as single agents or delivery ligands in molecular therapy (7). Nucleoside transporter–dependent, endosome-independent cellular penetration by a lupus anti-DNA autoantibody, 3E10, has previously been demonstrated (8–11). The key nucleoside transporter involved in cellular penetration by 3E10, ENT2, is widely expressed in cancer and normal cells. Notably, ENT2 in brain endothelial cells contributes to regulating nucleoside flux at the BBB, and a 3E10-heat shock protein fusion was previously shown to localize to and protect the ischemic brain (12–15). These findings raised the possibility that ENT2 may facilitate transport of 3E10 across the BBB and that 3E10 could be used to target brain tumors.

3E10 is not toxic to normal cells but is synthetically lethal to PTEN and BRCA-deficient cancer cells with defects in homologous recombination and repair of DNA double-strand breaks. Mechanistically, 3E10 inhibits base excision repair and homologous recombination and causes double-strand breaks to accumulate in and kill DNA repair-deficient cancer cells (8, 16–19). Furthermore, patterns of 3E10 tissue distribution after systemic administration confer a second layer of tumor specificity. 3E10 shows minimal uptake into normal tissues in healthy mice (20) but localizes to tumors or damaged tissues that release DNA into the local environment in animal models of malignancy, stroke, or myocardial infarction (10, 15, 21, 22). This relative targeting of tumors or damaged tissue is believed to result from the previously reported increase in efficiency of cellular penetration by 3E10 that is mediated by addition of extracellular DNA. DNA release by necrotic tumors or damaged tissues creates a DNA and nucleoside-rich environment that appears to attract 3E10 and facilitate its penetration into live cells that are salvaging nucleosides from the environment through ENT2. Based on this, we previously developed an autocatalytic method for use of 3E10 to target conjugated nanoparticles to tumors, where promotion of DNA release by dying tumors creates a positive feedback cycle that facilitates increasing localization of 3E10-decorated nanoparticles to tumors (21).

Necrosis is a defining feature of aggressive brain tumors such as glioblastoma multiforme (GBM) (23), and such tumors are compelling targets for 3E10. Furthermore, microRNAs liberated in the brain tumor microenvironment are suppressive of PTEN, and loss of PTEN function is common in primary and metastatic brain tumors (24–33) and yields an actionable impairment in homologous recombination that predicts vulnerability to 3E10 (8, 16–19, 34–37). The success of this approach is dependent on the ability of 3E10 to cross the BBB. In this mechanistic and proof-of-concept study, we investigated the ability of a reengineered and optimized fragment of 3E10, Deoxymab-1 (DX1), to penetrate brain endothelial cells and cross the BBB in an ENT2-dependent manner, and to target and suppress the growth of orthotopic brain tumors.

## Results

### ENT2 mediates brain endothelial cell penetration by a reengineered 3E10 fragment.

**Figure 1:**
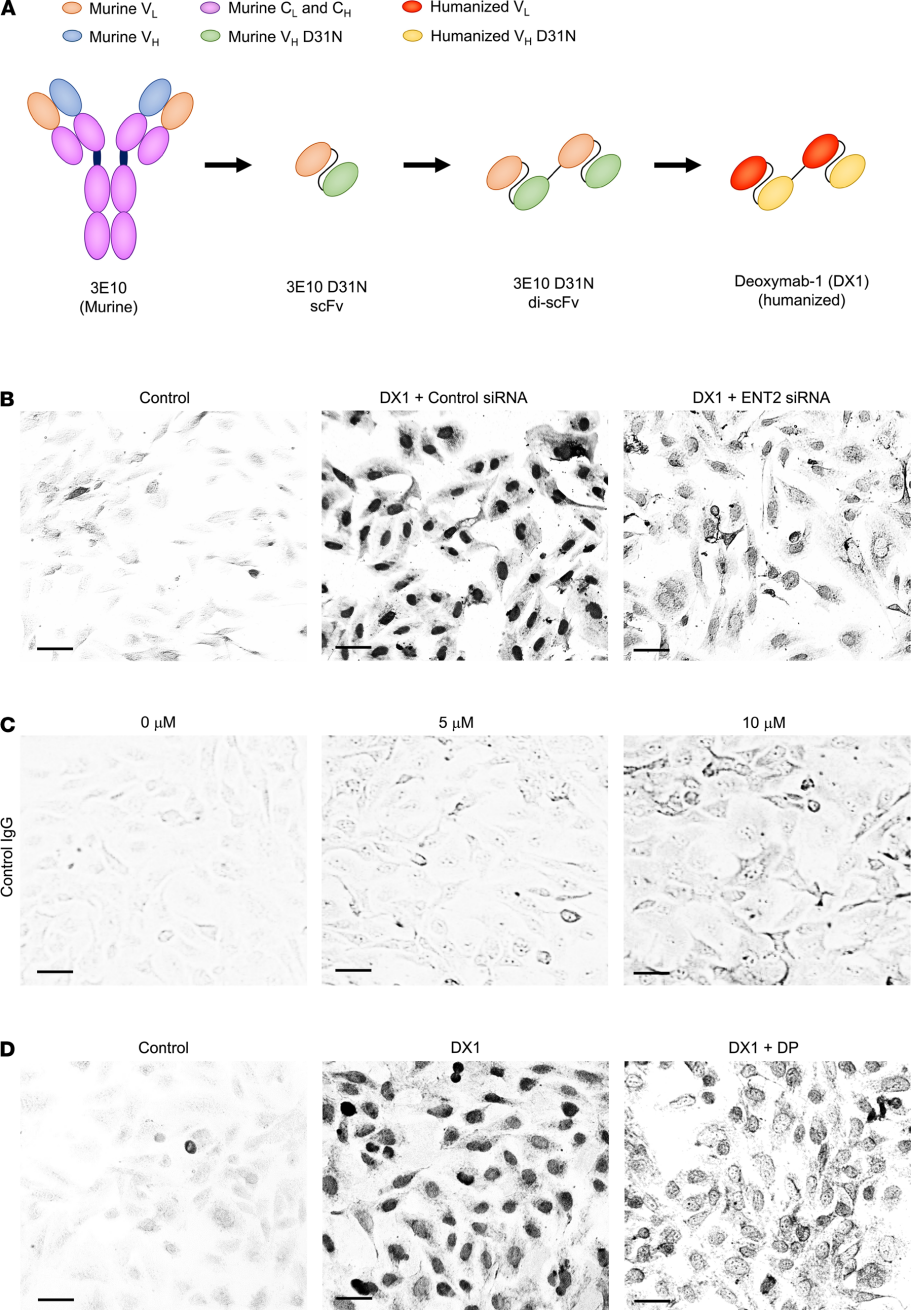
DX1 penetration into human brain endothelial cells is dependent on ENT2. (**A**) Illustrated evolution of 3E10 into DX1. 3E10 was isolated from the MRL/lpr lupus mouse model. A 3E10 single chain variable fragment (scFv) with D31N mutation in the heavy chain variable domain complementarity determining region 1 (V_H_ CDR1) was previously shown to have higher affinity for DNA and efficiency of cellular penetration compared with the original 3E10. 3E10 D31N di-scFv has greater impact on the DNA damage response and synthetic lethality to PTEN-deficient cancer cells due to its increased avidity for DNA compared with the scFv. The 3E10 D31N di-scFv was humanized, deimmunized, and CDR-optimized to yield DX1, which is now in development for testing in clinical trials (8, 17). (**B**) DX1 penetrates into brain endothelial cells in an ENT2-dependent manner. hCMEC/D3 cells transfected with control or ENT2-targeting siRNA were treated with control buffer or DX1 alone, and then stained to detect DX1 penetration. Representative images are shown. Scale bar: 30 μm. (**C**) Control IgG shows minimal uptake into brain endothelial cells. hCMEC/D3 cells were treated with 0–10 μM of a control IgG (specifically an anti-PD1 antibody) and stained to detect uptake of IgG. Representative images are shown. Scale bar: 30 μm. (**D**) The ENT2 inhibitor DP interferes with DX1 penetration into brain endothelial cells. hCMEC/D3 cells were treated with control buffer, DX1, or DX1 + 50 μM DP and stained for DX1. Representative images are shown. Scale bar: 30 μm. These data demonstrate ENT2-dependent penetration by DX1 into hCMEC/D3 cells.

hCMEC/D3 brain endothelial cells recapitulate intercellular junctions to restrict paracellular transport and are commonly used in transwell assays of BBB permeability (38, 39). We recently reengineered and optimized a 3E10 fragment to yield DX1, which is in development for clinical trial testing against multiple DNA repair–deficient tumors (Figure 1A) (8, 17). The Fc in 3E10 is not required for cellular penetration or its synthetically lethal effect on PTEN-deficient cancer cells, and in developing DX1, we have intentionally used the di-scFv structure that lacks an Fc in order to minimize risks of Fc-mediated off-target toxicity (8). ENT2 has been shown to be the key transporter that mediates uptake of 3E10 into other cell lines, but its role in DX1 penetration into brain endothelial cells has not previously been evaluated. The effect of ENT2 knockdown on efficiency of DX1 penetration into hCMEC/D3 cells was tested. Cells were transfected with control or ENT2-targeting siRNA, and ENT2 knockdown was confirmed by reverse transcription PCR (RT-PCR) (Supplemental Figure 1; supplemental material available online with this article; https://doi.org/10.1172/jci.insight.145875DS1). ENT2 knockdown reduced penetration by DX1 to 0.36 ± 0.04 (*P* < 0.01) relative to cells treated with DX1 and control siRNA (Figure 1B and Supplemental Table 1). Cells treated with control IgG showed minimal uptake of antibody (Figure 1C). These results demonstrate a role of ENT2 in the mechanism of DX1 penetration into hCMEC/D3 brain endothelial cells.

Equilibrative nucleoside transporters are sensitive to inhibition by the pyrimido-pyrimidine derivative drug dipyridamole (DP) (40, 41). The impact of DP on hCMEC/D3 penetration by DX1 was evaluated. Cotreatment with DP reduced penetration by DX1 into the cells to 0.41 ± 0.02 (*P* < 0.01) relative to cells treated with DX1 in the absence of DP (Figure 1D and Supplemental Table 2). These results are consistent with findings of the ENT2 knockdown experiment and further support ENT2-dependent penetration by DX1 into hCMEC/D3 cells. In addition, they identified DP as a viable drug for use in inhibiting DX1 penetration into brain endothelial cells in transwell insert assays and in vivo as described below.

### DX1 crosses a transwell model of the BBB in a DP-sensitive manner.

**Figure 2:**
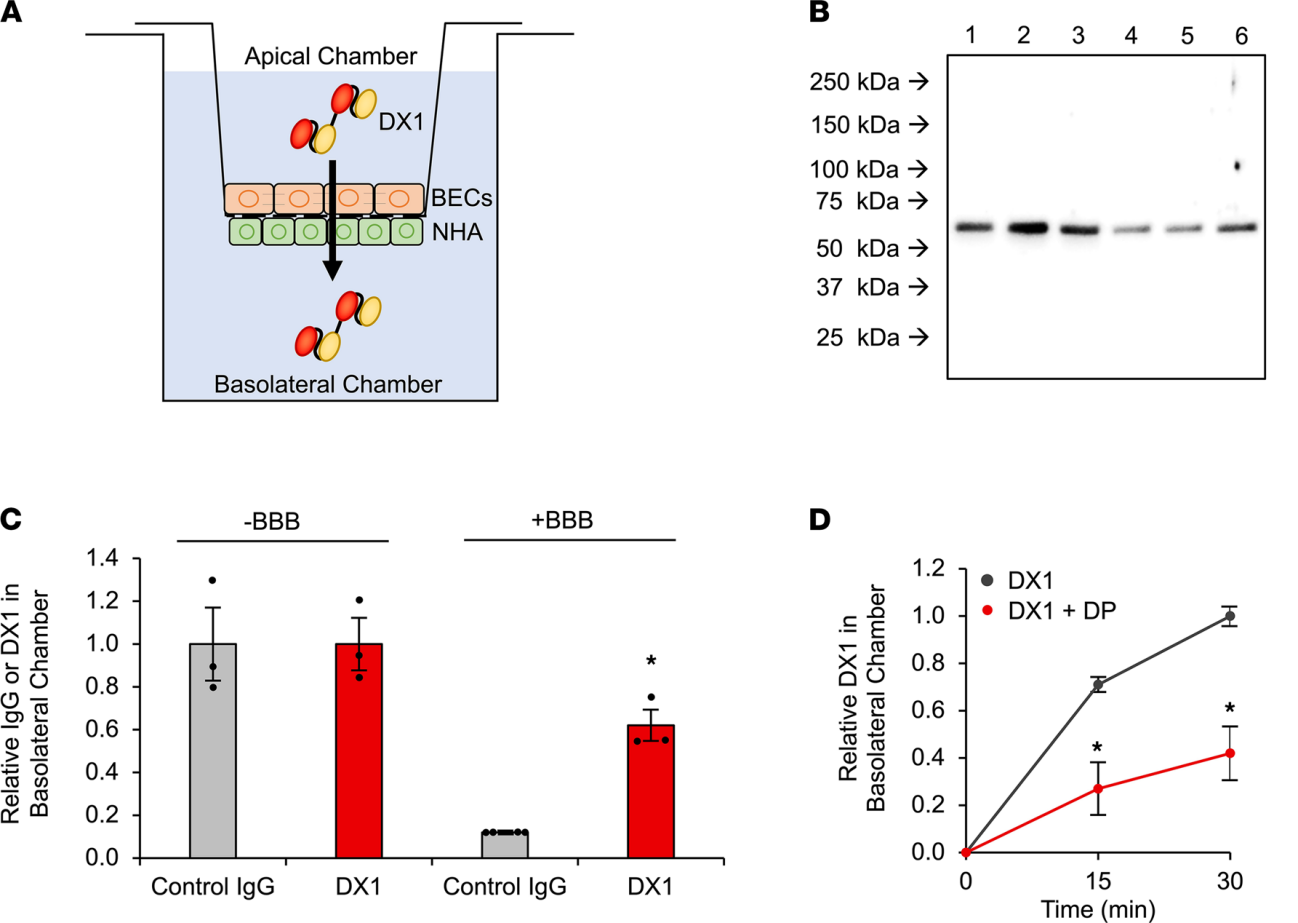
DX1 crosses a transwell model of the BBB in a DP-sensitive manner. (**A**) Illustrated transwell model used to test BBB crossing by antibodies. hCMEC/D3 BECs and normal human astrocytes (NHA) were adhered to apical and basolateral surfaces of transwell inserts, respectively. The ability of control IgG or DX1 to cross this model was tested by measuring their appearance in the basolateral chamber after addition to the apical chamber. (**B** and **C**) DX1 crosses the transwell model of the BBB. The efficiency of IgG and DX1 transport across control blank inserts and inserts with BBB was compared by anti-IgG or anti-DX1 Western blot of basolateral chamber contents 1 hour after addition of IgG or DX1 to apical chambers. Representative Western blot showing DX1 at expected molecular weight (~54 kDa) in basolateral chambers of blank inserts (lanes 1–3) and BBB inserts (lanes 4–6) is shown in **B**. Each lane represents an independent experiment. Control IgG blot is shown in Supplemental Figure 2. Presence of the BBB reduced the relative control IgG content in basolateral chambers to 0.12 ± 0.01 relative to chambers lacking the BBB, while DX1 content was only reduced to 0.62 ± 0.07 (*P* < 0.01 compared with control IgG, Student’s *t* test, *n* ≥ 3) as determined by ImageJ-based quantification of band intensities (**C**). (**D**) DP inhibits transport of DX1 across the hCMEC/D3 BBB. DX1 content in basolateral chambers was evaluated 15 and 30 minutes after addition of 5 μM DX1 ± 50 μM DP to apical chambers and quantified relative to DX1 content at the 30-minute time point in the absence of DP. *P < 0.01, Student’s *t* test, *n* = 3. These data demonstrate DP-sensitive DX1 transport across the hCMEC/D3 transwell BBB model, consistent with ENT2-dependent crossing of the BBB by DX1.

The ability of DX1 to cross an hCMEC/D3 transwell model of the BBB was tested using our previously described technique (39). Briefly, hCMEC/D3 cells were seeded onto apical sides of transwell inserts, and normal human astrocytes (NHA) seeded onto basolateral surfaces. Inserts were transferred to culture plates, establishing apical and basolateral chambers separated by the BBB model (Figure 2A). Tight junction formation on the inserts was visualized by occludin immunofluorescence, and successful establishment of functional barriers was verified by confirmation of expected transendothelial electrical resistance (TEER) and by demonstrating that the barrier-restricted movement of control BSA from apical to basolateral chambers (Supplemental Figure 2, A and B). DX1 content in basolateral chambers 1 hour after its addition to apical chambers in control blank inserts and BBB inserts was evaluated by anti-DX1 Western blot. DX1 successfully crossed the hCMEC/D3 BBB model, with basolateral content only reduced to 0.62 ± 0.07 (*P* < 0.03) in BBB inserts relative to control blank inserts (Figure 2, B and C). In contrast, presence of the BBB largely prevented control IgG from entering the basolateral chamber, with content reduced to 0.12 ± 0.01 relative to control blank inserts (*P* < 0.01 compared with DX1) (Figure 2C and Supplemental Figure 2C).

The siRNA-mediated approach to knockdown of ENT2 in the hCMEC/D3 cells was not compatible with the transwell model due to length of time required for barrier formation after transfection. The effect of DP on DX1 transport across the hCMEC/D3 BBB model was evaluated. DX1 content in basolateral chambers 15 and 30 minutes after its addition to apical chambers in the presence or absence of DP was measured, and fraction relative to DX1 content at 30 minutes in the absence of DP was calculated. In the absence of DP, the relative basolateral DX1 content reached 0.71 ± 0.03 and 1.0 ± 0.04 at 15 and 30 minutes, respectively. Cotreatment with 50 μM DP reduced relative basolateral DX1 contents to 0.27 ± 0.11 (*P* < 0.01) and 0.42 ± 0.11 (*P* < 0.01) at 15 and 30 minutes, respectively (Figure 2D). These results are consistent with ENT2-dependent crossing of the BBB by DX1.

### DP inhibits DX1 localization to orthotopic GBM tumors.

**Figure 3:**
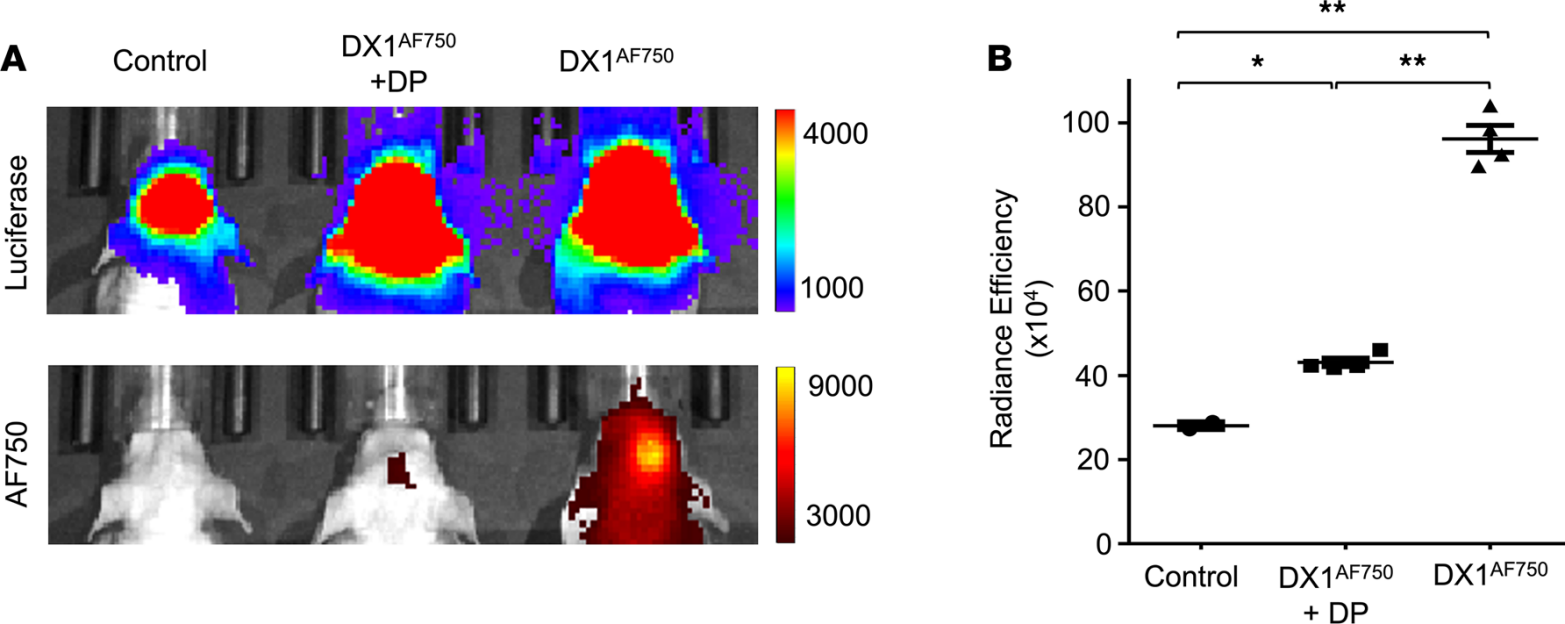
DX1 localizes to orthotopic GBM tumors in a DP-sensitive manner. Human GBM model 1 GSCs engineered to express luciferase were inoculated into the brains of immunodeficient mice (*n* = 10) to generate orthotopic PDX GBM tumors. (**A**) Representative IVIS images confirming presence of tumors (upper panel) and comparing DX1 localization to tumors (lower panel). DX1 was labeled with AF750 to facilitate its detection by IVIS. Mice were treated with i.v. and i.p. control buffer (*n* = 2), i.v. DX1^AF750^ (20 mg/kg) and i.p. control buffer (*n* = 4), or i.v. DX1^AF750^ (20 mg/kg) and i.p. DP (70 mg/kg) (*n* = 4). IVIS measurements 24 hours after treatment demonstrated DX1 localization into the tumors, and DP significantly suppressed this uptake. (**B**) Quantification of radiance efficiencies. *P = 0.0142, **P < 0.0001. Tukey’s multiple-comparison test–adjusted *P* values; numbers of mice evaluated at each point are indicated in the plots.

A panel of primary human GBM glioma stem-like cells (GSCs) derived from patient tumors is maintained at Yale School of Medicine under an IRB-approved protocol (42). GBM tumor model 1 GSCs from this panel have been engineered to express luciferase to allow tumor detection by IVIS Spectrum in vivo imaging system (IVIS). The ability of DX1 to localize to GBM tumor model 1 in the presence or absence of DP was evaluated. Immunodeficient mice inoculated intracranially with GBM tumor model 1 GSCs were evaluated by weekly IVIS to track luciferase signaling to confirm growth of brain tumors. Mice with established tumors (Figure 3A) were divided into groups for treatment with i.v. and i.p. injection of control buffer (*n* = 2), i.v. DX1 (20 mg/kg) and i.p. control buffer (*n* = 4), or i.v. DX1 (20 mg/kg) and i.p. DP (70 mg/kg) (*n* = 4). DX1 was labeled with Alexa Fluor 750 (AF750) to facilitate detection in vivo. Twenty-four hours after treatment, brains were evaluated by IVIS to detect the labeled DX1, and radiance efficiencies were recorded. Control mice showed a mean background radiance efficiency in the brain of 28.1 ± 0.9 *×* 10^4^. Mice treated with DX1 in the absence of DP exhibited a strong AF750 signal in the brain, correlating to the region of tumor formation, with mean radiance efficiency of 96.1 ± 3.2 × 10^4^ (Tukey’s multiple-comparison test adjusted *P* < 0.0001 relative to control mice). Cotreatment with DP reduced the observed uptake of DX1 into the brain tumors, with mean radiance efficiencies decreased to 43.1 ± 1.0 *×* 10^4^ (Tukey’s multiple-comparison test adjusted *P* < 0.0001 relative to mice treated with DX1 in the absence of DP). Representative images are shown in Figure 3A, and absolute radiance efficiencies are plotted in Figure 3B. Taken together, the findings in the transwell and orthotopic GBM tumor studies are consistent with DX1 crossing the BBB to localize into brain tumors in an ENT2-dependent manner.

### DX1 suppresses tumor growth and prolongs survival in orthotopic models of GBM.

**Figure 4:**
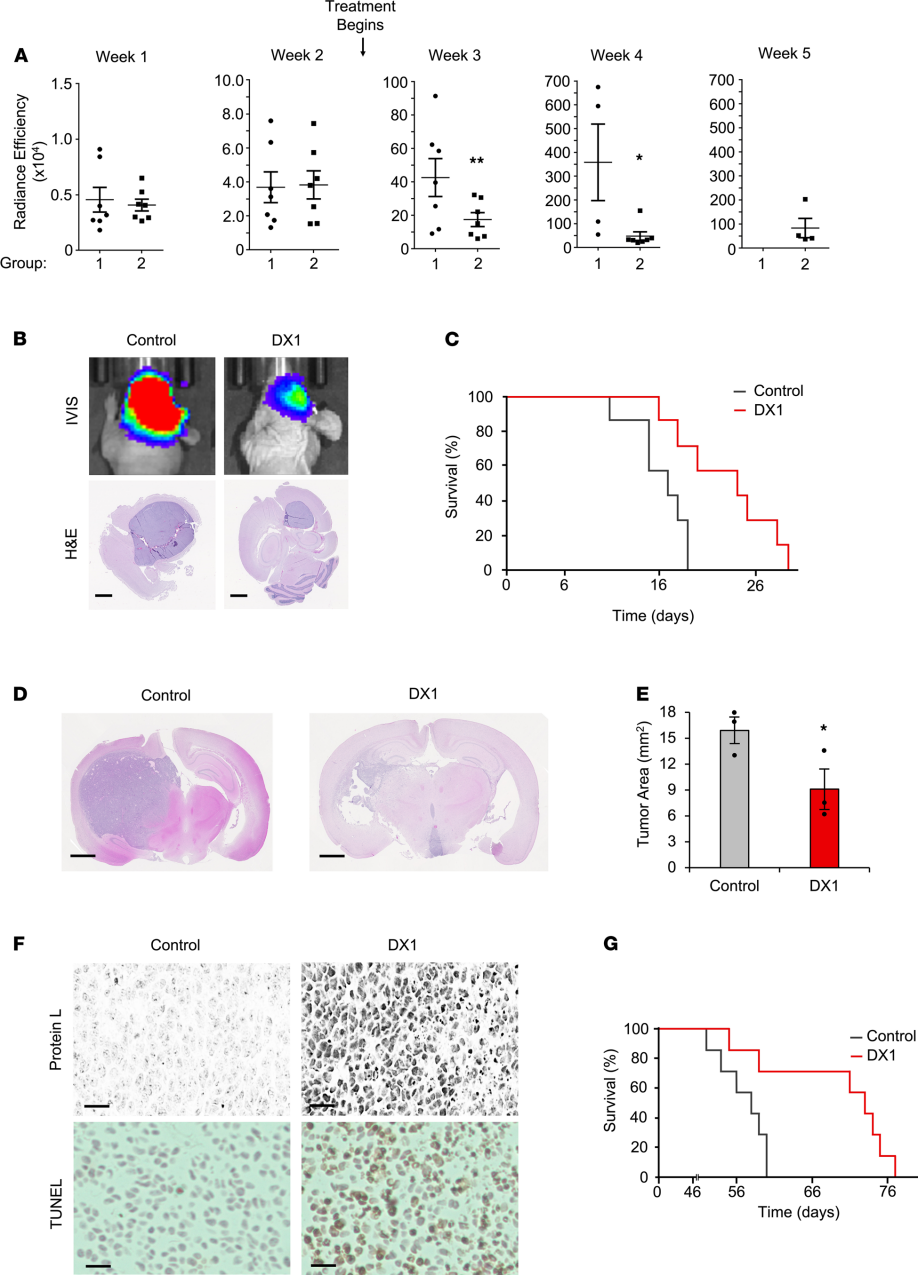
DX1 suppresses tumors in 2 orthotopic PDX models of GBM. (**A**–**C**) DX1 suppresses GBM model 1 tumors. Tumor growth after inoculation of luciferase-expressing GBM model 1 GSCs into the brains of immunodeficient mice was followed by radiance efficiency in the brain by weekly IVIS. Two weeks after inoculation, mice began treatment with i.v. control buffer (group 1) or DX1 (20 mg/kg, group 2). (**A**) Radiance efficiencies are shown and demonstrate significant suppression of tumors by DX1 (note the *y* axis scale increases over the weeks). **P < 0.04, Student’s *t* test, *n* = 7 per group. *P < 0.02, Student’s *t* test, *n* = 4 and 7 per group. (**B**) Representative IVIS images and H&E stains are shown. Scale bar: 1.25 mm. (**C**) Treatment with DX1 increased median survival (measured from initiation of treatment) to 24 days, compared with 17 days in control mice (*P* < 0.01, log-rank test, *n* = 7). (**D**–**G**) DX1 suppresses GBM model 2 tumors. Two weeks after inoculation of GBM model 2 GSCs, mice began treatment with i.v. control buffer (*n* = 10) or 20 mg/kg DX1 (*n* = 10) 3 times per week. At 9 weeks after inoculation, brains from 3 mice per group were analyzed by H&E and Ki67 stains to facilitate measurement of mean tumor areas. (**D** and **E**) Representative H&E sections and mean tumor areas (*P = 0.04, Student’s *t* test, *n* = 3). Scale bar: 1.25 mm. (**F**) Sections of tumors from mice treated with control or DX1 stained to detect DX1 with protein L are shown and demonstrate that DX1 crossed the BBB to penetrate tumors and was associated with increased TUNEL staining. Scale bar: 50 μm. Results were confirmed by separately staining tumor sections with an anti-DX1 antibody (Supplemental Figure 4). (**G**) DX1 prolonged median survival in GBM model 2 to 73 days, compared with 58 days in mice treated with control (*P* = 0.02, log-rank test, *n* = 7).

As previously discussed, 3E10 is synthetically lethal to PTEN-deficient cancer cells (8, 16–19). Approximately 90% of GBM exhibits loss of heterozygosity involving the PTEN locus, and 30%–40% of primary GBM carries a secondary somatic PTEN mutation (24–27). The GBM tumor model 1 GSCs used in Figure 3 form PTEN-deficient tumors when inoculated into the brains of immunodeficient mice, and DX1 was confirmed to suppress their spheroid growth in culture (Supplemental Figure 3). To determine the effect of DX1 on tumor growth in vivo, mice inoculated with GSCs were randomized to group 1 for treatment with i.v. control buffer (*n* = 7) or group 2 for treatment with i.v. DX1 (20 mg/kg) (*n* = 7) 3 times per week throughout the study. Consistent tumor growth between groups was confirmed by IVIS prior to treatment. One week after inoculation, mean radiance efficiencies (**×** 10^4^) ± SEM in groups 1 and 2 were 0.5 ± 0.1 and 0.4 ± 0.1, respectively (*P* = 0.36, *n* = 7 per group). At 2 weeks after inoculation, mean radiance efficiencies (**×** 10^4^) ± SEM in groups 1 and 2 were 3.7 ± 0.9 and 3.8 ± 0.8, respectively (*P* = 0.46, *n* = 7 per group). Treatment began after the week 2 IVIS measurements. At week 3 (1 week after the start of treatment), treatment with DX1 was observed to suppress tumor growth, with radiance efficiencies (**×** 10^4^) in groups 1 and 2 of 42.6 ± 11.1 and 17.4 ± 4.1, respectively (*P* < 0.04, *n* = 7 per group). In week 4, radiance efficiencies (**×** 10^4^) in groups 1 and 2 were 358.1 ± 160.9 and 47.7 ± 17.7, respectively (*P* < 0.02, *n* = 4 and 7) (Figure 4, A and B). By week 5 all group 1 mice had expired, while 57% (4 out of 7) of mice in group 2 were alive. Overall, DX1 was well tolerated (Supplemental Figure 4) and prolonged the median survival (measured from initiation of treatment) from 17 to 24 days (*P* < 0.01, *n* = 7 mice per group, log-rank test) (Figure 4C).

DX1 was tested in a second orthotopic GBM model, GBM model 2. GBM model 2 GSCs from the same panel of patient-derived tumor cells maintained at Yale School of Medicine were confirmed to form PTEN-deficient tumors in the brains of immunodeficient mice, and comparison of spheroid volumes after treatment with control or DX1 demonstrated significant suppression of spheroid growth by DX1 in a dose-dependent manner (Supplemental Figure 3). The ability of DX1 to localize to and suppress orthotopic GBM model 2 tumors was evaluated. GSCs were inoculated into the brains of immunodeficient mice, and 2 weeks later, mice began treatment with i.v. (by tail vein injection) control buffer (PBS, *n* = 10) or DX1 (20 mg/kg, *n* = 10) 3 times per week throughout the study. GBM model 2 GSCs have not been engineered to express luciferase, which necessitated examination of the effect of DX1 on tumor growth by harvesting brains at a preselected time point. At 9 weeks after tumor inoculation, 3 mice per group were euthanized, and brains were sectioned for analysis by H&E, Ki67, and TUNEL stains and immunostained for DX1. This time point was selected based on experience with this model in regard to time of onset of symptoms. Tumor area in each brain was evaluated by importing images of H&E- and Ki67-stained sections into ImageJ for contouring, and measurement of mean tumor areas on slices through the maximal thickness of the tumor. Representative H&E and Ki67 sections are shown in Figure 4D and Supplemental Figure 4. Mean tumor areas were reduced in mice treated with DX1 compared with control (9.1 ± 2.3 mm^2^ versus 15.9 ± 1.5 mm^2^, *P* < 0.04, *n* = 3 mice per group) (Figure 4E). Both protein L and anti-DX1–based immunostaining detected DX1, which demonstrated DX1 transport across the BBB into the tumors (Figure 4F and Supplemental Figure 4). In addition, DX1 was associated with increased tumor TUNEL staining (Figure 4F). DX1 was not detected in the uninvolved brain remote from the tumor (Supplemental Figure 4). The remaining mice continued treatment, and DX1 increased median survival (measured from initiation of treatment) to 73 days, compared with 58 days in control mice (*P* = 0.02, *n* = 7 mice per group, log-rank test) (Figure 4G). DX1 was well tolerated, with no significant difference in mean body weights between control and DX1 groups throughout the study (Supplemental Figure 4). These studies demonstrate DX1 tumor penetration and suppression in 2 separate orthotopic PDX models of GBM.

### DX1 suppresses tumor growth and prolongs survival in an orthotopic model of breast cancer brain metastases.

**Figure 5:**
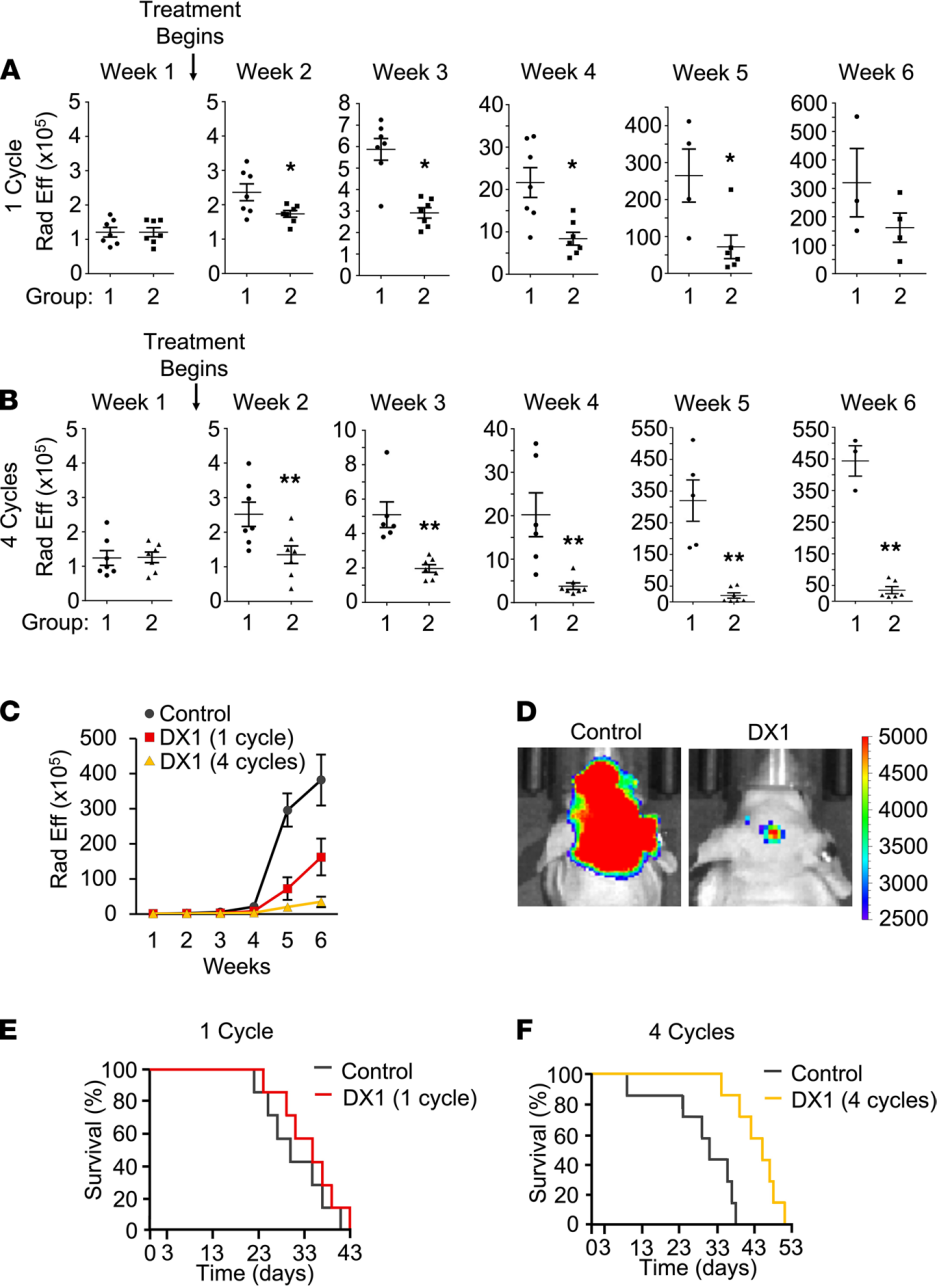
DX1 suppresses tumor growth and prolongs survival in a 231-BR orthotopic model of breast cancer brain metastases. Formation and growth of brain metastases in mice after intracardiac injection of luciferase-expressing 231-BR cells was followed by monitoring radiance efficiency in the brain. One week after injection, mice began treatment with i.v. control buffer (group 1) or DX1 (20 mg/kg, group 2). Weekly IVIS demonstrated significant suppression of tumor growth by DX1 delivered as 1 or 4 cycles. (**A**–**D**) Radiance efficiencies are plotted (**A**–**C**), and representative IVIS images from week 5 of the 4 cycle study are shown (**D**). *P < 0.04, **P ≤ 0.01. Student’s *t* test; numbers of mice evaluated at each point are indicated in the plots. (**E**) One cycle of DX1 was associated with a nonsignificant increase in median survival to 35 days, compared with 30 days in control mice (*P* = 0.42, log-rank test, *n* = 7). (**F**) Four cycles of DX1 had a greater effect, with median survival prolonged to 45 days, compared with 31 days in control mice (*P* < 0.02, log-rank test, *n* = 7).

Breast cancer brain metastases are associated with increased incidence of PTEN and homologous recombination defects compared with primary breast tumors (28–33). The brain-seeking subclone of the MDA-MB-231 breast cancer cell line, MDA-MB-231-BR (43) (hereafter referred to as 231-BR), has been reported to exhibit loss of PTEN expression (44) compared with parental cells. 231-BR cells yield multiple metastatic brain lesions after intracardiac injection, representing one of the most challenging brain tumor models to treat (45). DX1 was confirmed to penetrate and reduce clonogenic survival of 231-BR cells in colony formation assays (Supplemental Figure 5), and the effect of DX1 on 231-BR brain metastases in vivo was evaluated. Brain metastases were generated in immunodeficient mice by intracardiac injection of 231-BR cells engineered for expression of luciferase. Brain metastases were confirmed by IVIS 1 week after injection, and mice were randomized to group 1 to receive i.v. control buffer (PBS, *n* = 7) or group 2 for i.v. DX1 (20 mg/kg, *n* = 7, group 2). The effects of DX1 delivered as a single cycle or as 4 consecutive cycles were tested in 2 independent studies, with 1 cycle defined as administration of control or DX1 3 times in 1 week. Mice were observed for behavior and weights, and brain radiance efficiency was monitored by weekly IVIS to track tumor growth. For the single cycle study, at week 1 prior to the start of treatment, mean radiance efficiencies (× 10^5^) ± SEM in groups 1 and 2 were 1.2 ± 0.1 and 1.2 ± 0.1, respectively (*P* = 0.47, *n* = 7 per group). Treatment with DX1 suppressed tumor growth, with radiance efficiencies (× 10^5^) in groups 1 and 2 of: 2.4 ± 0.2 and 1.7 ± 0.1 in week 2 (*P* < 0.03, *n* = 7 per group), 5.9 ± 0.6 and 2.9 ± 0.3 in week 3 (*P* < 0.01, *n* = 7 per group), 21.6 ± 3.5 and 8.4 ± 1.5 in week 4 (*P* < 0.01, *n* = 7 per group), 264.8 ± 72.0 and 71.9 ± 31.3 in week 5 (*P* < 0.04, *n* = 4 and 6), and 319.9 ± 122.5 and 161.8 ± 51.4 in week 6 (*P* = 0.16, *n* = 3 and 4), respectively (Figure 5A). For the 4 cycle study, at week 1 prior to the start of treatment, mean radiance efficiencies (× 10^5^) ± SEM in groups 1 and 2 were 1.2 ± 0.2 and 1.3 ± 0.2, respectively (*P* = 0.48, *n* = 7 per group). Treatment with DX1 suppressed tumor growth, with radiance efficiencies (× 10^5^) in groups 1 and 2 of: 2.5 ± 0.4 and 1.4 ± 0.3 in week 2 (*P* ≤ 0.01, *n* = 7 per group), 5.1 ± 0.7 and 2.0 ± 0.2 in week 3 (*P* ≤ 0.01, *n* = 6 and 7), 20.2 ± 5 and 3.8 ± 0.8 in week 4 (*P* ≤ 0.01, *n* = 6 and 7), 320 ± 66 and 20.2 ± 8.5 in week 5 (*P* ≤ 0.01, *n* = 5 and 7), and 444 ± 49 and 35.3 ± 11 in week 6 (*P* ≤ 0.01, *n* = 3 and 6), respectively (Figure 5B). Radiance efficiencies for the 2 studies compared with combined controls from each study demonstrate a high level of reproducibility of this brain metastasis model (Figure 5, C and D). One cycle of DX1 yielded a nonsignificant increase in median survival (measured from initiation of treatment) from 30 to 35 days (*P* = 0.42) (Figure 5E), similar to previous studies of paclitaxel in this model (46). Four cycles of DX1 had a greater impact, with median survival from initiation of treatment increased by 14 days (from 31 to 45) (*P* < 0.02) (Figure 5F). DX1 was not associated with significant behavior changes or weight loss compared with controls (Supplemental Figure 5). These data demonstrate the ability of DX1 to suppress tumor growth and improve survival in an orthotopic model of breast cancer brain metastases, and they indicate that duration of DX1 treatment contributes to the magnitude of response.

## Discussion

Antibody-based immunotherapy has the potential to change paradigms in the management of CNS malignancies, but obstacles inherent to the neuroimmune interface must be overcome. The BBB excludes most antibodies and likely contributes to the suboptimal responses of primary and metastatic brain tumors to antibodies observed in clinical trials to date (47–49). The autoimmune disease SLE may offer a solution to this problem. In the present study, we found that DX1, a re-engineered fragment of a lupus autoantibody that penetrates cells in an ENT2-dependent manner, crosses an intact BBB in a transwell model and localizes to and suppresses the growth of tumors in orthotopic GBM and brain metastasis models. The precise mechanism by which DX1 is transported through the BBB remains to be elucidated, but our present findings implicate the nucleoside transporter ENT2 as a key factor.

In previous work, cellular and nuclear penetration by 3E10 was demonstrated in multiple cell lines and shown dependent on the ability of 3E10 to bind DNA, the presence of extracellular DNA/nucleosides, and the expression of ENT2 in cells (9, 10, 50–52). Most conclusively, a 3E10 scFv was unable to penetrate ENT2-deficient cells but rapidly transduced cells with restored expression of ENT2 (9). In the present study, we found that siRNA-mediated knockdown of ENT2 reduced penetration by DX1 into brain endothelial cells, and this finding further strengthens the hypothesis that ENT2 is a key transporter in this process.

The pyrimido-pyrimidine derivative drug DP was used to facilitate consideration of the role of ENT2 in DX1 transport across a transwell BBB model and into brain tumors in vivo. DP blocked DX1 penetration into brain endothelial cells, transport across the transwell BBB model, and localization into orthotopic brain tumors. These findings further implicate ENT2 as mediator of BBB transport and brain tumor targeting by DX1. In regard to specificity of DP activity, DP exhibits greater inhibition of ENT2 as compared with the prototype nucleoside transport inhibitor nitrobenzyl mercaptopurine riboside (NBMPR). DP also has inhibitory effects on equilibrative nucleoside transporters ENT1 and ENT4 and on phosphodiesterases (53–56). Based on our previously published work with 3E10 in cells differentially expressing various nucleoside transporters (9), as well as the ENT2 knockdown results obtained in the present work, we are confident that the impact of DP on DX1 penetration into brain endothelial cells and transport across the BBB is due to its inhibitory effects on ENT2 function. Additional studies including tissue-specific KO of nucleoside transporters in mice are planned for confirmation of these findings. Furthermore, the details of the interaction between DX1 and ENT2 and the mechanisms that control trafficking of DX1 into nuclei and/or out of cells into basolateral chambers remain to be determined.

Taken together, our present findings support an ENT2-mediated mechanism of BBB penetration by DX1 and establish proof of concept for the use of a DNA-targeting lupus autoantibody against brain tumors. We also recognize DX1 as a potential delivery ligand for targeting linked cargo molecules to brain tumors and as a platform upon which additional antibodies with CNS bioavailability may be designed, including DX1-based bispecific antibodies. Furthermore, our findings raise the possibility that nucleoside salvage transporters are involved in the crossing of the BBB by neurotoxic autoantibodies in the syndrome of CNS lupus (57, 58) and that drugs such as DP may suppress the uptake of these autoantibodies into the brain to help ameliorate CNS pathology. While this is speculative, it is notable that DP has previously been shown to improve outcomes in a mouse model of lupus (59), and results of a clinical trial of DP in SLE are pending (NCT01781611). Lupus autoantibodies that hijack nucleoside transporters to cross cell membranes represent potentially actionable targets in SLE and new frontiers in brain tumor therapy.

## Methods

### Antibodies and cells.

DX1 (PAT-DX1, Patrys Ltd.) was expressed in CHO cells and purified over a HiTrap Capto S column by FPLC as previously described (17). Purity and quality was confirmed by SDS-PAGE and SEC-HPLC prior to use. DX1 concentrations were determined by Bradford assay. The control IgG used in brain endothelial cell penetration and transwell experiments was rat IgG2a anti-PD1 and was obtained from Leinco (catalog P372). Pierce Recombinant Protein L (catalog 21189), anti–protein L (catalog PA-72066), anti-occludin (catalog OC-3F10), alkaline phosphatase (AP), horse radish peroxidase (HRP), and Alexa 488–conjugated secondary antibodies (catalogs 31350, 31470, and A28175) were obtained from Thermo Fisher Scientific. Rat primary anti-DX1 antibody (clone D5) was provided by Patrys Ltd. and was used at 1:1000 in cell and tissue staining and immunoblots. Human GSCs were obtained from a panel of GSCs harvested from primary GBM tumors resected at Yale School of Medicine under an IRB-approved protocol (42). Cells were maintained in Neurobasal culture medium (Thermo Fisher Scientific) supplemented with bFGF (20 ng/mL, Peprotech, 100-18B), EGF (20 ng/mL, Peprotech, 100-15), penicillin-streptomycin-glutamine (Thermo Fisher Scientific, 10378016), and B27 (Thermo Fisher Scientific, 12587-010). 231-BR cells were provided by T. Yoneda (Osaka University, Suita, Japan) (43). hCMEC/D3 brain endothelial cells (catalog SCC066) were obtained from MilliporeSigma and NHA from Lonza. Unless otherwise specified, all other cell culture reagents and media were obtained from Thermo Fisher Scientific.

### Brain endothelial cell penetration assay.

DP (D9766) was obtained from MilliporeSigma. hCMEC/D3 BECs were cultured in 96-well plates and were then pretreated with control buffer (PBS + 10% FBS) or buffer containing 50 μM DP for 1 hour. Supernatants were then replaced with control buffer, buffer containing 10 μM DX1, or buffer containing 50 μM DP and 10 μM DX1. Thirty minutes later, cells were washed with PBS 3 times, fixed with chilled ethanol, and immunostained with the D5 anti-DX1 primary antibody (1:1000, Patrys Ltd.), anti–rat AP-–conjugated secondary antibody (1:2000, catalog 31350, Thermo Fisher Scientific), and finally with DX1 signal detected by color development as previously described (17). Cell staining intensity was quantified by ImageJ (NIH). The hCMEC/D3 cells were also separately treated with control buffer or control IgG (0–10 μM) for 30 minutes and then washed, fixed, and immunostained for IgG with anti–rat AP–conjugated secondary antibody (catalog 31350, Thermo Fisher Scientific), with color development as described above.

### ENT2 knockdown.

hCMEC/D3 cells were grown to 50% confluence. In total, 60 nM ENT2 siRNA pool (siGENOME SMARTpool, Horizon) or nontargeting control siRNA (Dharmacon, Horizon) were transfected into the cells using Lipofectamine RNAiMAX reagent (Thermo Fisher Scientific) according to the manufacturer’s instructions. The transfection was repeated the next day to ensure maximum knockdown. Successful ENT2 knockdown was confirmed by RT-PCR. RNA from ENT2 siRNA- and control siRNA–transfected knock down hCMEC/D3 cells was extracted using RNeasy kit (Qiagen) according to the manufacturer’s instructions. RNA was reverse transcribed into cDNA using QuantiTect Reverse Transcription Kit (Qiagen) according to the manufacturer’s protocol using 0.5 μg RNA in a total of 20 μL reaction. The mRNA level of ENT2 and β-actin was assessed using TaqMan Gene Expression real-time PCR assays (TaqMan probe; Hs01546959_g1 and Hs0160665_g1, respectively; Applied Biosystems). The results were expressed as the Ct. The relative quantification of the target transcripts normalized to the endogenous control β-actin was determined by the comparative Ct method (ΔCt), and the 2^–ΔΔCt^ method was used to analyze the relative changes in gene expression between the tested samples according to the manufacturer’s protocol (User Bulletin No. 2, Applied Biosystems). After confirmation of ENT2 knockdown, efficiency of DX1 penetration into control or ENT2 siRNA–transfected cells was compared by protein L-based staining as previously described (17).

### BBB transwell model.

Cell culture inserts (24-well format, 0.4 μm, PET track-etched membrane, 353095, MilliporeSigma) were coated with 0.001% poly-L-lysine (PLL) solution (diluted in sterile distilled water, P4832, MilliporeSigma) on the basolateral side and Fibronectin solution (diluted in sterile PBS, F1141, MilliporeSigma) on the apical side. NHA were adhered to the basolateral side of inserts (4.5 *×* 10^4^ cells/insert), and hCMEC/D3 brain endothelial cells were adhered to the apical side (3.3 *×* 10^4^ cells/insert).

### Immunostaining for occludin.

Tight junctions between the hCMEC/D3 cells on the inserts were evaluated by immunostaining for occludin. Cells were fixed in chilled 100% ethanol for 10 minutes, and inserts were then washed with PBS (950 μL in basolateral and 250 μL in apical chambers) and blocked with 20% goat serum/0.5% Triton X-100 in PBS. The inserts were then transferred to wells containing 0.5% BSA/0.25% Triton X-100 in PBS (antibody incubation buffer [AIB]). The apical chamber contents were replaced with primary antibody against occludin (1:20) in AIB. Inserts were incubated at room temperature for 1 hour and then washed 3 times using PBS containing 0.1% Triton X-100 (950 μL in basolateral and 250 μL in apical chambers for each wash). Alexa 488–conjugated secondary antibody (1:200) in AIB was then added to apical chambers, and AIB to basolateral chambers, for 1 hour at room temperature. The inserts were subsequently washed with PBS-T as mentioned above. DAPI (Thermo Fisher Scientific, D3571) counterstain was then completed by addition of DAPI in PBS to apical chambers for 10 minutes (along with PBS alone to basolateral chambers), followed by washes. All steps were conducted on inserts still attached to culture plate adaptors until final washes were completed. Inserts were then gently excised from the adaptors using a scalpel and mounted onto glass slides using Aqua Poly/Mount mounting medium to facilitate visualization of fluorescence by Evos FL microscope (Thermo Fisher Scientific). The slides were kept at 4°C overnight and imaged the next day.

### Testing the integrity of the BBB on the inserts.

TEER of inserts with BBB (with both hCMEC/D3 and NHA layers of cells) and control blank inserts (with only PLL and Fibronectin) was serially measured by voltometer, and when the difference was more than 100, the BBB inserts were deemed ready to be used in experiments (39, 60). To test the ability of a control IgG or DX1 to cross the transwell BBB model, 5 μM control IgG or DX1 in PBS supplemented with 10% FBS was applied to the apical chambers in control blank inserts (–BBB) or inserts with the BBB (+BBB) for 1 hour; then, control IgG or DX1 content in basolateral chambers was determined by anti-IgG or anti-DX1 Western blot. Control IgG or DX1 content in the basolateral chambers of BBB inserts compared with control blank inserts was then determined by ImageJ quantification of results. As an additional negative control, BSA labeled with Alexa Fluor 555 (555-BSA, A34786, Thermo Fisher Scientific) was similarly applied to apical chambers, and content in basolateral chambers 1 hour later was evaluated by a fluorescence plate reader (Synergy HT, BioTek). To test the impact of DP on DX1 crossing of the transwell BBB model, transwell BBB models were treated with control buffer or buffer containing 50 μM DP for 30 minutes, after which 5 μM DX1 ± 50 μM DP was added to apical chambers as described above. Aliquots from the basolateral chamber (50 μL) were taken at 15 and 30 minutes and analyzed by anti-DX1 dot blot, and DX1 content — relative to the 30-minute sample in the absence of DP — was determined by ImageJ.

### Testing localization by DX1 ± DP to orthotopic GBM.

Studies were conducted under a Yale University IACUC–approved protocol. In the first model, luciferase-expressing GBM model 1 human GSCs were used to establish orthotopic GBM tumors in the brains of nude mice. Female athymic NCr-nu/nu mice (5–6 weeks old, Charles River Laboratories) maintained in a pathogen-free environment were anesthetized by i.p. injection of ketamine and xylazine (Covetrus). In total, 6 *×* 10^4^ GSCs in 5 μL PBS were injected into the right striatum (*n* = 10) 2 mm lateral and 0.5 mm posterior to the bregma and 3 mm below the dura, using a stereotactic apparatus with an UltraMicroPump (UMP3) (World Precision Instruments). Mice were then tracked by weekly IVIS (IVIS Spectrum, PerkinElmer), and once tumors were confirmed, mice were randomized to groups for treatment with tail vein and i.p. injection of control buffer (PBS for tail vein, DMSO/PEG 400/PBS for i.p.) (*n* = 2), tail vein injection of DX1^AF750^ (20 mg/kg, in PBS) and i.p. injection of control buffer (*n* = 4), or tail vein injection of DX1^AF750^ (20 mg/kg, in PBS) and i.p. injection of DP (70 mg/kg, in DMSO/PEG 400/PBS) (*n* = 4). DX1 was labeled with equimolar AF750 by reaction with AF750 NHS Ester (Thermo Fisher Scientific) in PBS (pH 7.4). AF750-labeled DX1 was dialyzed (Snakeskin 3.5K MWCO, Thermo Fisher Scientific) into PBS to remove excess AF750, and it was filter-sterilized prior to use. Twenty-four hours after treatment, mice were evaluated by IVIS to detect AF750 signal in the brain tumors.

### Spheroid volume assay.

Volumes of GBM spheroids treated with control media or media containing DX1 were monitored over 7 days. An Evos FL microscope (Thermo Fisher Scientific) was used to capture representative bright-field images over multiple fields of view. Analysis of GBM spheroid volume was performed using ImageJ. Sphere images were imported, and the scale was thresholded to calibrate for pixel size. Sphere diameters were determined and used to determine the equivalent sphere volume for each spheroid. Relative volumes were calculated as described in each experiment.

### Colony formation assay.

The impact of control media or media containing DX1 on the clonogenic survival of 231-BR cells was evaluated by colony formation assay as previously described (16).

### Efficacy studies in orthotopic GBM models.

Studies were conducted under a Yale University IACUC–approved protocol. Two separate GSCs (referred to as GBM models 1 and 2) were used to establish orthotopic GBM tumors in the brains of nude mice. PTEN-deficient status of tumors was confirmed by anti-PTEN immunostain on sections of established tumors as previously described (10). Female athymic NCr-nu/nu mice (5–6 weeks old, Charles River Laboratories) maintained in a pathogen-free environment were anesthetized by i.p. injection of ketamine and xylazine. In total, 5 *×* 10^4^ to 6 *×* 10^4^ GSCs in 5 μL PBS were injected into the right striatum (*n* = 14 for the first model study, *n* = 20 for the second model study), 2 mm lateral and 0.5 mm posterior to the bregma and 3 mm below the dura, using a stereotactic apparatus with an UltraMicroPump (UMP3) on day zero. Treatment was initiated 2 weeks after inoculation. Mice were treated with control buffer (PBS, *n* = 7 and 10 for GBM models 1 and 2, respectively) or DX1 (20 mg/kg, *n* = 7 and 10 for GBM models 1 and 2, respectively) by tail vein injection 3 times per week. Mice were monitored daily for general health, body weight, grooming, behavioral changes and any signs of distress. Mice exhibiting neurological symptoms or significant weight loss were humanely euthanized. In the GBM model 1 study, tumor sizes were tracked by weekly IVIS as previously described (39). For the GBM model 2 study in which cells were not engineered to express luciferase, at week 9 after inoculation, 3 mice per group were sacrificed for evaluation of tumor size and localization of DX1 into the brain tumors. Brains were harvested, formalin-fixed, and embedded in paraffin. Sections were analyzed by H&E, Ki67, and TUNEL stain, and by anti-DX1 and protein L-based immunostaining to detect DX1 using previously described protocols (10, 21). The remaining 7 mice per group were observed until neurological symptoms and/or weight loss were noted, and they were humanely euthanized. For both of the GBM model studies, Kaplan-Meier plots of survival were generated in GraphPad Prism (version 7).

### Breast cancer brain metastases model.

Studies were conducted under a Yale University IACUC–approved protocol. The 231-BR brain-seeking subclone of the MDA-MB-231 cell line, engineered to express luciferase to allow detection by IVIS, was used to establish brain metastases in nude mice. Two independent studies with this model were conducted in order to test the effects of a single cycle or 4 cycles of DX1. In each study, on day zero, 14 female athymic NCr-nu/nu mice (5–6 weeks old, Charles River Laboratories) maintained in a pathogen-free environment were anesthetized by i.p. injection of ketamine and xylazine, and they were subjected to intracardiac injection of 1.75 *×* 10^5^ 231-BR cells in 100 μL PBS. Successful establishment of brain metastases was confirmed by IVIS 1 week after injection. Mice were randomized into groups with equivalent mean radiance efficiencies in the brain, and they were treated with control vehicle (PBS, *n* = 7) or DX1 (20 mg/kg, in PBS, *n* = 7) by tail vein injection 3 times per week for 1 or 4 cycles. Mice were monitored daily for general health, body weight, grooming, behavioral changes, and any signs of distress. Mice exhibiting neurological symptoms or significant weight loss were humanely euthanized. Tumor burden was monitored by weekly IVIS. Kaplan-Meier plots of survival were generated in GraphPad Prism (version 7).

### Statistics.

Log-rank test was used to determine the statistical significance of in vivo survival data. Tukey’s multiple-comparison test was used to determine the statistical significance of radiance efficiency data in Figure 3B. For the remainder of the measurements, a 1-tailed Student’s *t* test was used to evaluate the statistical significance of data. *P* ≤ 0.05 was considered significant. Data are presented as mean ± SEM in figure parts in which error bars are shown. Mice were randomized to respective treatment groups. Sample sizes were selected based on power analysis informed by previous results with the parental 3E10 antibody and fragments. Endpoints were prospectively selected. Number of replicates for each experiment are indicated in the respective figures. No investigator blinding was used.

### Study approval.

Studies were conducted under a Yale University IACUC–approved protocol.

## Author contributions

Study conception and design were contributed by JZ and JEH. Performance of experiments and analysis of data were contributed by ZR, GD, SZ, AS, CKM, XC, BJC, PZ, JL, NJWR, JZ, and JEH. Manuscript preparation and/or review were contributed by ZR, GD, SZ, AS, CKM, XC, BJC, JL, PZ, NJWR, CHJ, VD, JAC, AH, JMB, JZ, and JEH. The order of the co–first authors (ZR, GD, and SZ) was based on the timing of each author’s initiation of their work on the project.

## Supplementary Material

Supplemental data

## Version 1 06/15/2021

In-Press Preview

## Version 2 07/22/2021

Electronic publication
