# Supplementary material for: ENT2 facilitates brain endothelial cell penetration and blood-brain barrier transport by a tumor-targeting anti-DNA autoantibody
Source: JCI Insight. 2021 Jul 22;6(14):e145875. doi: 10.1172/jci.insight.145875 (PMC8410084; doi:10.1172/jci.insight.145875)
Supplement: Supplemental data [file jciinsight-6-145875-s060.pdf]

Supplementary Material

Figure S1

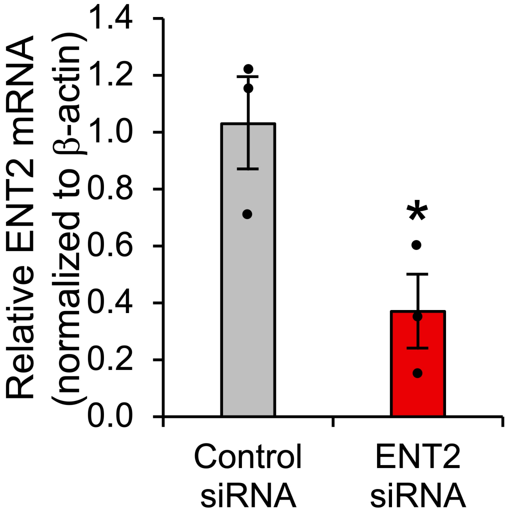

Figure S1. Confirmation of ENT2 knockdown. RT-PCR confirmation of siRNA-mediated ENT2 knockdown in hCMEC/D3 cells. RNA from ENT2 siRNA- and control siRNA-transfected hCMEC/D3 cells was extracted and reverse transcribed into cDNA, and mRNA level of ENT2 and β-actin was assessed using TaqMan Gene Expression real-time PCR assay. Relative quantification of the target transcripts normalized to the endogenous control β-actin was determined by the comparative Ct method (ΔCt) and the 2-ΔΔCt method to analyze the relative changes in gene expression. *P<0.04, Student's t-test, n=3.

Figure S2

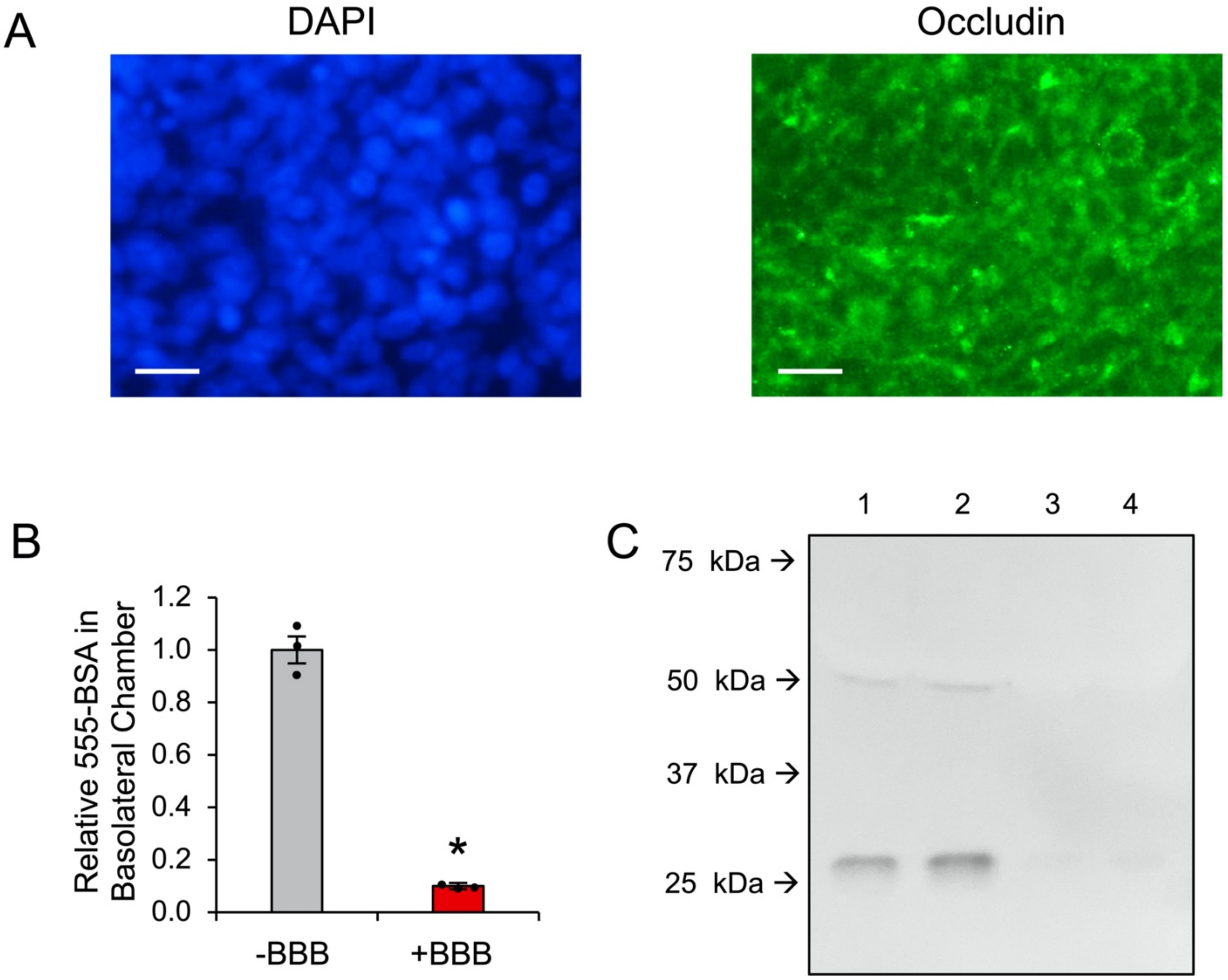

Figure S2. Confirmation of hCMEC/D3 BBB formation. (**A**) Representative DAPI and occludin immunofluorescence images in hCMEC/D3 cells on transwell filter. Bar = 20 μm. (**B**) hCMEC/D3 BBB restricts movement of BSA from apical to basolateral chambers. The efficiency of BSA transport across control blank filters (-BBB) and filters with BBB (+BBB) was compared by measuring Alexa Fluor 555 (AF555) fluorescence in basolateral chambers one hour after addition of AF555-labeled BSA to apical chambers. Presence of the BBB reduced the relative 555-BSA content in basolateral chambers to 0.10±0.01 (*P<0.01, Student's t-test, n=3) relative to chambers lacking the BBB. (**C**) hCMEC/D3

BBB restricts movement of a control IgG from apical to basolateral chambers. Control IgG content in basolateral chambers one hour after addition to apical chambers was measured by western blot. Representative western blot is shown. Lanes 1 and 2: IgG content in basolateral chamber in absence of BBB. Lanes 3 and 4: IgG content in basolateral chamber in presence of BBB. Observed bands correlate to expected IgG heavy and light chains.

Figure S3

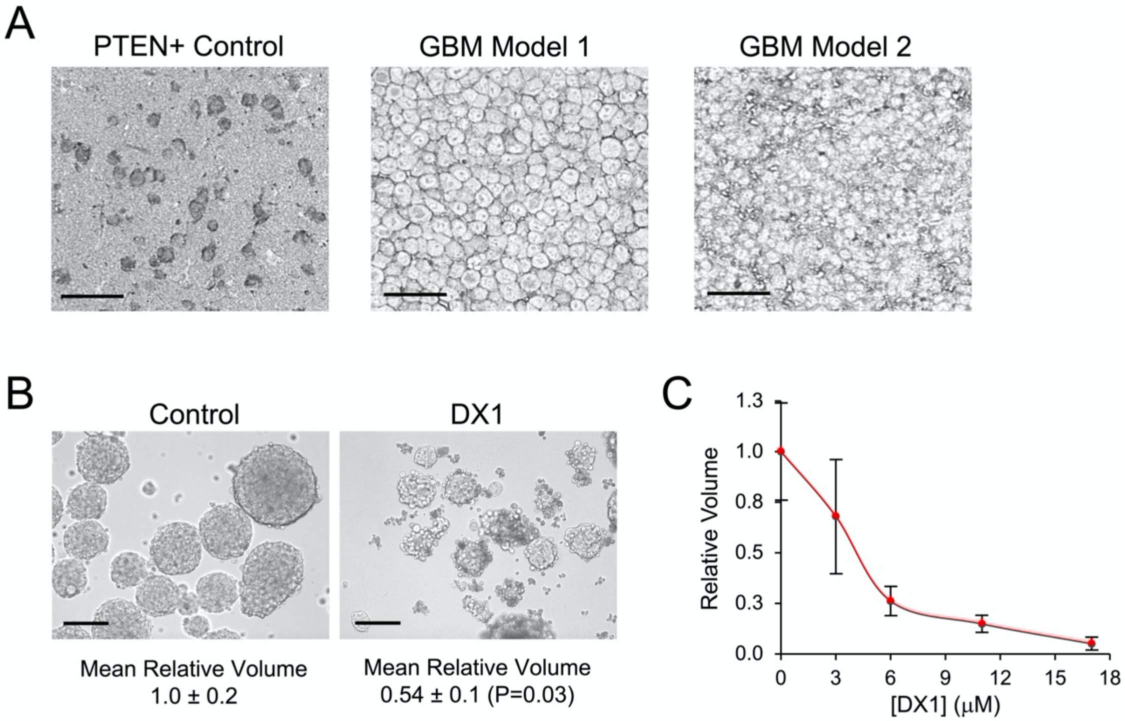

Figure S3. Orthotopic PDX GBM models 1 and 2 are negative for PTEN by IHC, and DX1 suppresses their spheroid growth in culture. (**A**) Sections of normal brain and GBM model 1 and 2 grown in the brains of immunodeficient mice were immunostained for PTEN. Normal brain stained positive for PTEN as expected, while both orthotopic GBM tumors stained negative. Bar = 50 μm. (**B**) DX1 inhibits GBM model 1 spheroid growth. Cultured spheroids were treated with control media or media containing 10 μM DX1, and relative volumes calculated one week later. Representative brightfield images of spheroids and mean relative volumes ± SEM (based on measurements of minimum of 30 spheroids in each group) are shown in (**B**). Bar = 100 μm. Student's t-test used for P value. (**C**) DX1 inhibits GBM model 2 spheroid growth in a dose-dependent manner. Cultured spheroids were treated with control media or media containing 0-17 μM DX1,

and relative volumes ± SEM calculated (based on measurements of minimum of 8 spheroids at each dose) one week later.

Figure S4

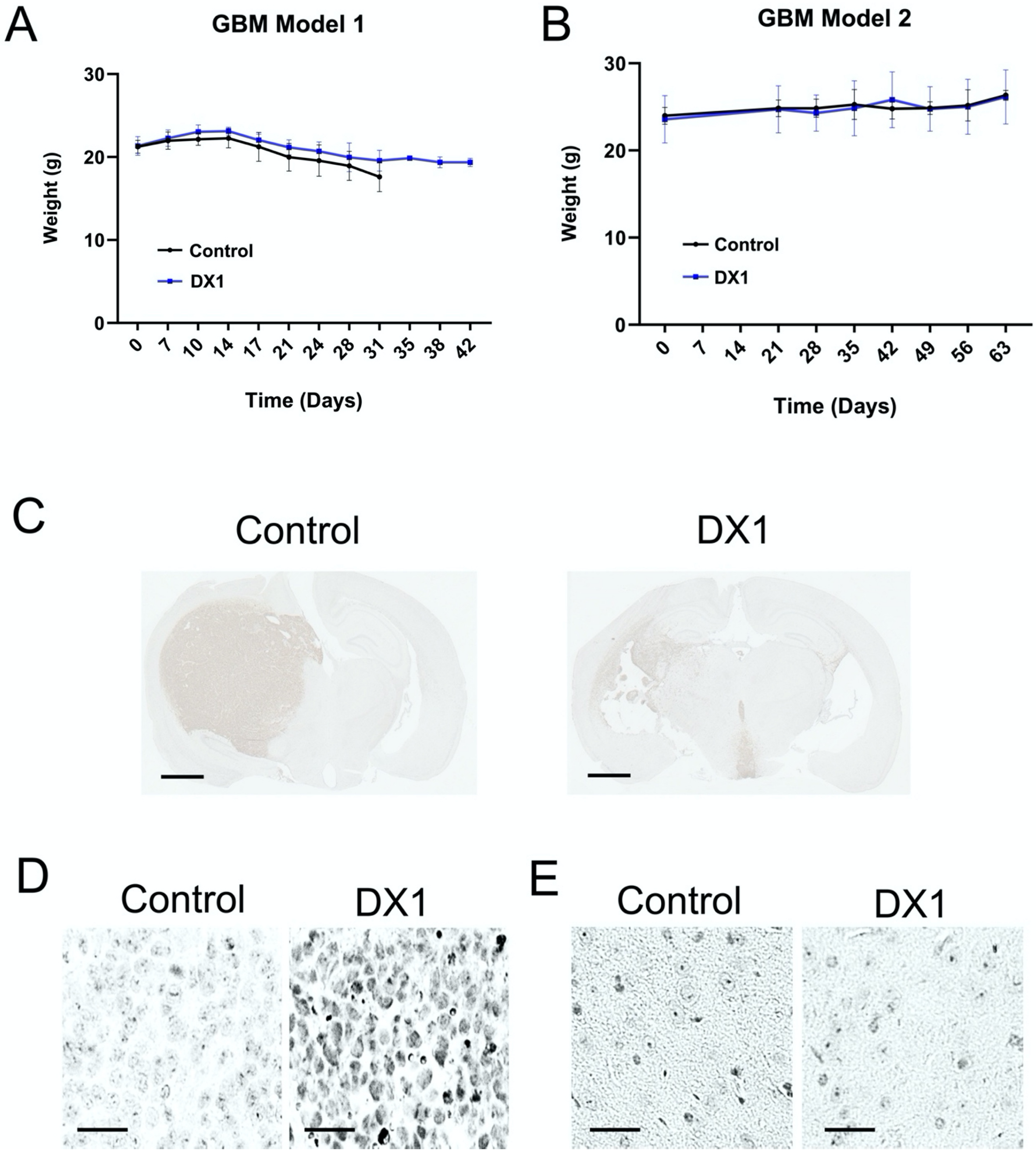
A
GBM Model 1
30
20
10
0
Weight (g)
Control
DX1
0 7 10 14 17 21 24 28 31 35 38 42
Time (Days)
B
GBM Model 2
30
20
10
0
Weight (g)
Control
DX1
0 7 14 21 28 35 42 49 56 63
Time (Days)
C
Control
DX1
D
Control
DX1
E
Control
DX1

Figure S4. Orthotopic GBM studies. (**A, B**) DX1 was well-tolerated in GBM model 1 and 2. Mean body weights in mice treated with control or DX1 are shown. (**C**) Representative images of Ki67-stained sections of tumors from GBM model 2 treated with control or DX1. Bar = 1.25 mm. (**D**) DX1 crosses the BBB to penetrate orthotopic GBM tumors. Representative sections of GBM model 2 tumors from mice treated with control or DX1 stained for DX1 with an anti-DX1 primary antibody are shown. DX1 was detected in the tumors, consistent with the protein L-based staining results shown in **Fig. 4F**. Bar = 50 μm. (**E**) DX1 is not detected in normal brain tissue. Representative images of protein L-based immunostained sections of brain remote from tumors in mice with orthotopic GBM model 2 tumors treated with control or DX1 are shown. Absence of staining in both sections demonstrates minimal uptake of DX1 into normal brain. Bar = 50 μm.

Figure S5

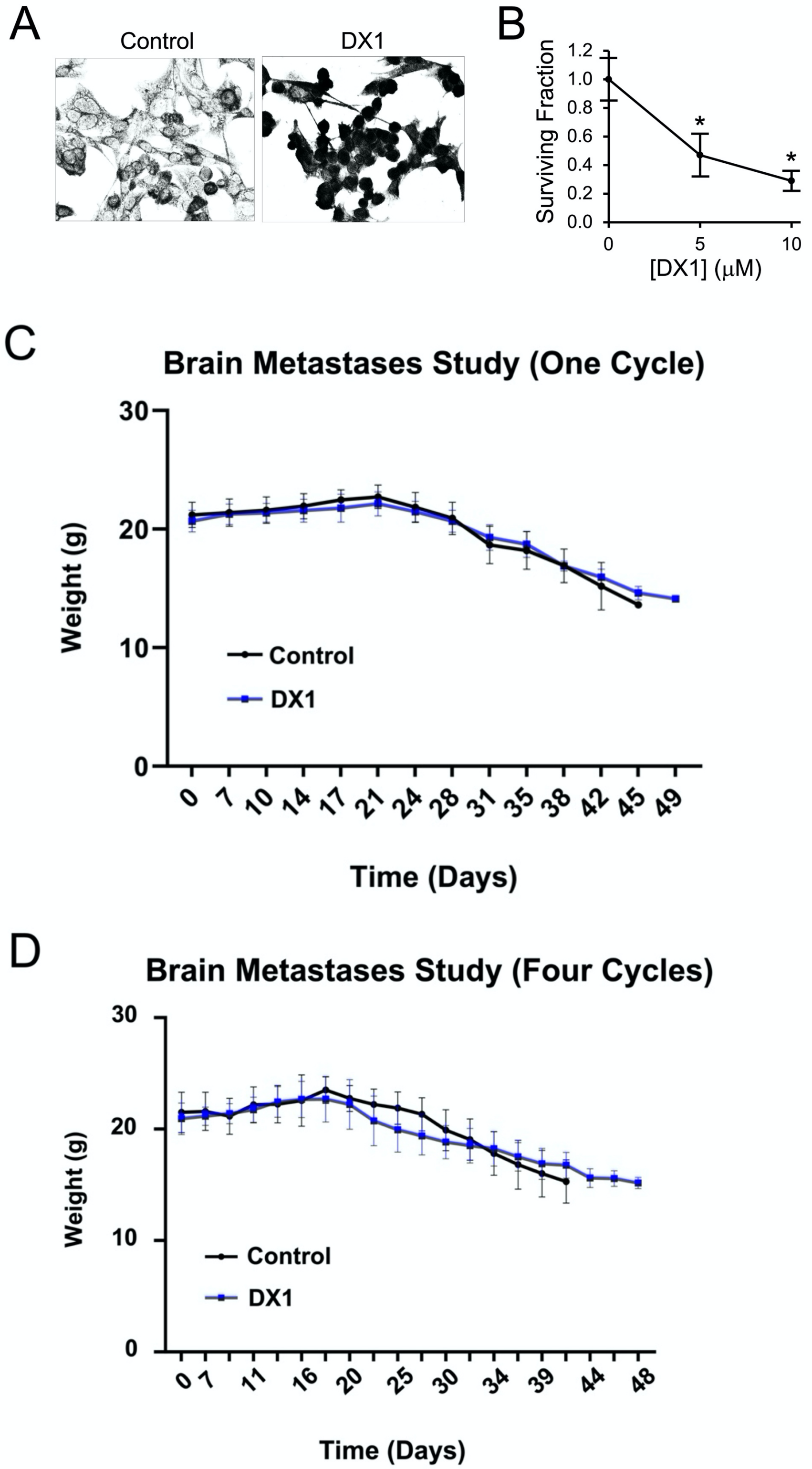
A
Control
DX1
B
Surviving Fraction
1.2
1.0
0.8
0.6
0.4
0.2
0.0
0
5
10
[DX1] (μM)
*
*
C
Brain Metastases Study (One Cycle)
Weight (g)
30
20
10
0
Control
DX1
0
7
10
14
17
21
24
28
31
35
38
42
45
49
Time (Days)
D
Brain Metastases Study (Four Cycles)
Weight (g)
30
20
10
0
Control
DX1
0
7
11
16
20
25
30
34
39
44
48
Time (Days)

Figure S5. DX1 penetrates and is toxic to 231-BR cells. (**A**) 231-BR cells were treated with control or 10 μM DX1 for one hour, and then washed, fixed, and subjected to protein L-based immunostaining. Representative images of stained cells are shown. (**B**) Colony formation assays evaluated the effect of DX1 on 231-BR clonogenic survival, and doses of 5 and 10 μM DX1 reduced surviving fractions to 0.47±0.15 (P<0.03, Student's t-test, n=3) and 0.29±0.07 (P<0.01, Student's t-test, n=3), respectively. (**C, D**) DX1 was well-tolerated in the brain metastases studies. Mean body weights in mice treated with control or DX1 in the brain metastases studies are shown.

Table S1

ENT2 Knockdown	
**Treatment**	**Relative Nuclear Staining**
DX1 + Control siRNA	1.00 ± 0.04
DX1 + ENT2 siRNA	0.36 ± 0.04 (P<0.01)

Table S1. ENT2 knockdown suppresses cellular and nuclear penetration by DX1. Mean nuclear staining intensity ± SEM is shown and reflects measurements of a minimum of 20 cells in each condition and is representative of three independent experiments. Student's t-test used for P value.

Table S2

DP (ENT2 Inhibitor)	
**Treatment**	**Relative Nuclear Staining**
DX1 + Control Buffer	1.00 ± 0.04
DX1 + DP	0.41 ± 0.02 (P<0.01)

Table S2. The ENT2 inhibitor DP suppress cellular and nuclear penetration by DX1. Mean nuclear staining intensity ± SEM is shown and reflects measurements of a minimum of 20 cells in each condition and is representative of three independent experiments. Student's t-test used for P value.
